# Rare Radiological Pattern of Diffuse Esophageal Spasm

**DOI:** 10.5811/westjem.2015.3.25826

**Published:** 2015-04-02

**Authors:** Demis N. Lipe, Nathan Borden

**Affiliations:** * Martin Army Community Hospital, Department of Emergency Medicine, Fort Benning, Georgia; † Carl R. Darnall Army Medical Center, Department of Emergency Medicine, Fort Hood, Texas

A 42-year-old man with history of esophageal strictures and esophageal dilation presented to the emergency department with 12 hours of dysphagia and non-bloody emesis. His symptoms started upon waking and included sharp retrosternal pain during each attempt at swallowing. Dysphagia occurred with both solids and liquid. He denied difficulty initiating swallowing, pain with eating the previous night, halitosis and hematemesis. His vitals and 12-lead electrocardiogram were normal. Despite several attempts at esophageal relaxation using standard methods, he continued having symptoms. At this point an esophagram was obtained (Figure).

The esophagram shows a dilated proximal esophagus and grossly abnormal mid-esophagus with abrupt cutoff of ingested barium contrast. The differential diagnosis of these findings is broad and includes Chagas disease, malignancy and obstruction. An esophagealduodenoscopy was ordered; however, the patient’s symptoms abruptly resolved without additional intervention.

This case illustrates an uncommon radiological finding in likely diffuse esophageal spasm (DES). The classic radiographic pattern, resulting from strong muscular contractions, resulting in near-complete lumen obliteration is not present, and there is no evidence for the classic fluoroscopic appearance of a “corkscrew” or “rosary bead” esophagus.^1^ The small contractions of the proximal esophagus in this case do not obliterate most of the lumen. This image supports several studies showing that barium studies in DES are usually not characterized by a corkscrew appearance.^1^–^4^ These studies illustrate that radiography alone is insufficient to diagnose DES. However, since DES occurs intermittently, it is a difficult diagnosis to make as esophagram and manometry cannot be performed together.^3^ Although our patient had a classic presentation, he did not have the classic radiographic finding. This image further illustrates the non-specific information provided by a barium swallow and importance of history and physical exam in the diagnosis of diffuse esophageal spasm.

## Figures and Tables

**Figure:**
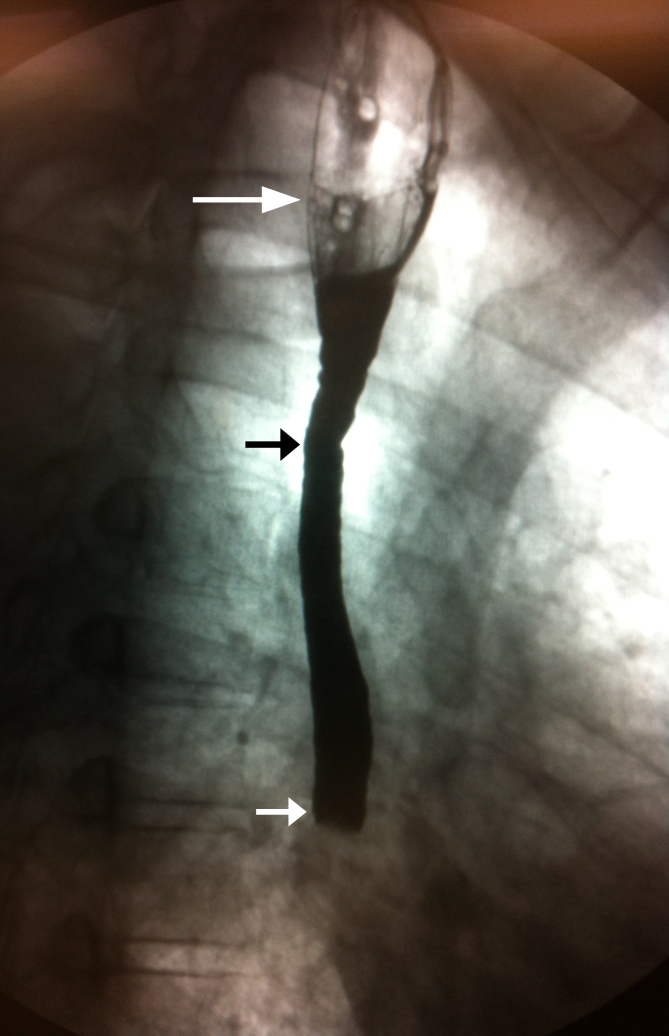
Barium esophagram. Grossly abnormal esophagram with dilated proximal esophagus (white arrow), abnormal appearance of middle one-third (short black arrow) and abrupt cutoff of ingested barium contrast material (short white arrow).
